# Cardiac Arrest (CA) as the Initial Presentation of Cocaine-Induced Takotsubo Cardiomyopathy (TCM): A Case Report and Review of Literature

**DOI:** 10.7759/cureus.38525

**Published:** 2023-05-04

**Authors:** Catherina Almalouf, Narek Hakobyan, Vivek Yadav, Anjula Gandhi, Ruchi Yadav

**Affiliations:** 1 Internal Medicine, Brookdale University Hospital Medical Center, Brooklyn, USA; 2 Internal Medicine, St. George's University School of Medicine, Brooklyn, USA; 3 Pulmonary and Critical Care, State University of New York Downstate Health Sciences University, New York, USA

**Keywords:** cardiovascular disease, takotsubo's syndrome, takotsubo cardiomyopathy, cocaine, cardiac arrest

## Abstract

Cocaine is used as an illicit substance responsible for the most common cause of drug-related death. It is a stimulant that acts on the sympathetic nervous system and cardiovascular system leading to exaggerated, prolonged sympathetic activity due to the accumulation of neurotransmitters. Cardiovascular side effects of cocaine are coronary artery spasms, myocarditis, arrhythmias, and congestive heart failure. Takotsubo cardiomyopathy (TCM) is characterized by transient hypokinesis, akinesis, or dyskinesis of the left ventricle (LV) wall with or without apical involvement in the absence of obstructive coronary artery disease. Cocaine-induced TCM is an extremely rare condition emphasizing the need of its prompt diagnosis by the physicians. We present a case report of a 54-year-old male, brought to the emergency department (ED) after an out-of-hospital cardiac arrest (CA), found to have TCM in the setting of cocaine use. Clinicians need to understand the association between cocaine use and the development of TCM as cardiomyopathy of this type can result in complete remission after discontinuing the offending agent.

## Introduction

More than 500,000 patients present to the ED every year with cocaine-associated complications including chest pain and approximately 40% of all ED visits, related to drug abuse, were attributed to cocaine use [1-2]. Cocaine is a sympathomimetic agent that acts by inhibiting the reuptake of norepinephrine, dopamine, and serotonin through binding with each transporter resulting in an accumulation of these neurotransmitters in the postsynaptic terminal. This leads to an exaggerated, prolonged sympathetic nervous system activity [3-4]. Cocaine has a direct effect on the cardiovascular system by stimulating alpha- and beta 1-adrenoceptors leading to increased heart rate, systemic arterial pressure, and myocardial contractility, all of which are major causative factors of myocardial oxygen demand [5]. Takotsubo syndrome (TTS), also called TCM, is a rare disease with a prevalence of only 0.5%-0.9% in the general population [6]. TCM is characterized by transient left ventricular wall dysfunction that is often triggered by physical or emotional stressors and often misdiagnosed as acute coronary syndrome [7]. Cocaine-induced TCM is even rarer as to date only a few case reports have been documented [8-13]. Herein, we present a unique case report of a 54-year-old male who developed Takotsubo cardiomyopathy (TCM) following recent cocaine use presenting with cardiac arrest (CA).

## Case presentation

A 54-year-old male with past medical history of morbid obesity, bipolar disorder, diabetes mellitus, and alcohol use presented to the emergency department (ED) after an out-of-hospital CA. His downtime was unknown. Cardiopulmonary resuscitation (CPR) was performed, and 4 mg of Naloxone was given on-site outside the hospital. The initial pulse was regained by the time emergency medical services (EMS) arrived, but it was lost shortly after. He was found to be in ventricular fibrillation (VF) and two shocks were given along with 300 mg of amiodarone and two doses of epinephrine. Return to spontaneous circulation (ROSC) was achieved after approximately 10 min. At the time of arrival in the ED, the patient was intubated, and medical staff performed a second set of CPR after he was found to have pulseless electrical activity (PEA). He was given an additional 150 mg of amiodarone, lidocaine, and started on epinephrine drip. ROSC were achieved 10 min after an initial rhythm of VF. The total time of resuscitation was approximately 20 min including CPR by EMS and ED staff.

**Figure 1:**
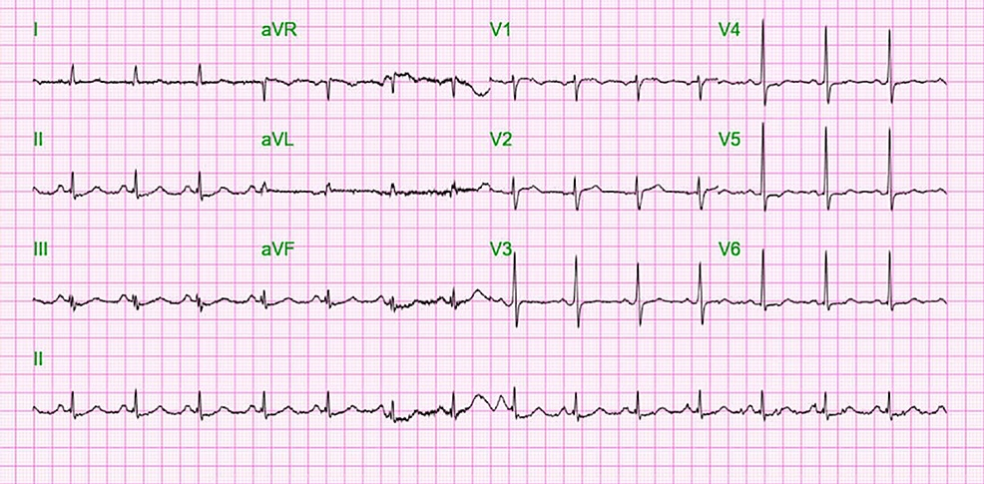
ECG showing normal sinus rhythm with nonspecific ST and T wave abnormalities. ECG, electrocardiogram

On admission, an arterial blood gas (done on inspired fraction of oxygen-100%) showed severe metabolic acidosis with pH 7.29 [reference range (ref) 7.35-7.45], pCO2 41.3 mmHg (ref: 35.0-45.0 mmHg), pO2 196 mmHg (ref: 80.0-110.0 mmHg), and HCO3 10.2 mmol/L (ref: 22.0-26.0 mmol/L). Bloodwork was significant for elevated high sensitivity troponin at 5,559 pg/mL (ref: <5.0-19.7 pg/mL), lactate of 11.4 mmol/L (ref: 0.70-2.10 mmol/L), d-dimer of 36,342 ng/mL (ref: <=230 ng/mL), blood urea nitrogen 8.0 mg/dL (ref: 9.0-20.0 mg/dL), creatinine 1.30 mg/dL (ref: 0.66-1.25 mg/dL), creatinine kinase total 254 U/L (ref: 55.00-170.00 U/L), white blood cell count 25.1 10 x 3/uL (ref: 4.1-10.1 10 x 3/uL), and a negative blood culture. A urine toxicology screening revealed the presence of cocaine. The level of alcohol in the blood was <10.00 mg/dL (ref: 0.00-10.00 mg/dL). On head CT, he was found to have a diffuse anoxic brain injury. After stabilizing, the patient had a blood pressure of 124/90 mmHg, a pulse rate of 95 bpm, and was afebrile. A normal sinus rhythm was detected on the electrocardiogram (ECG), with nonspecific ST and T wave abnormalities (Figure 1).

**Figure 2:**
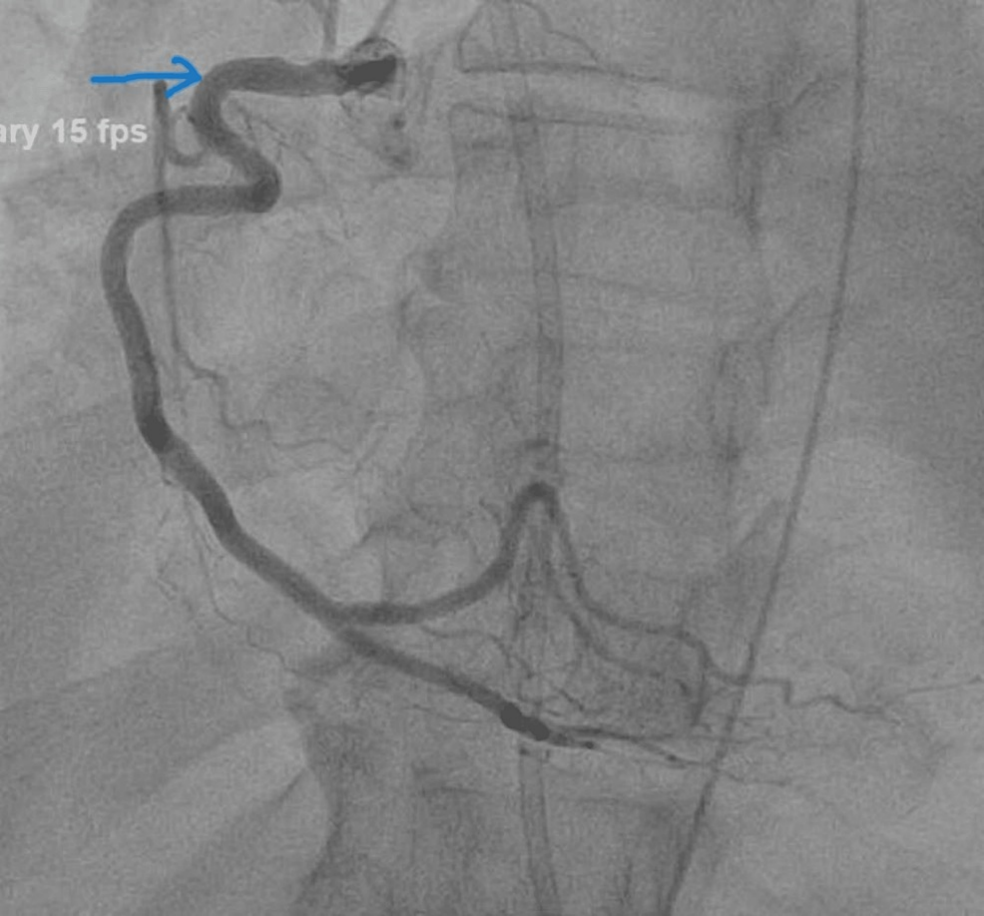
Coronary angiography showing flow in RCA with no evidence of obstruction. RCA, right coronary artery

**Figure 3:**
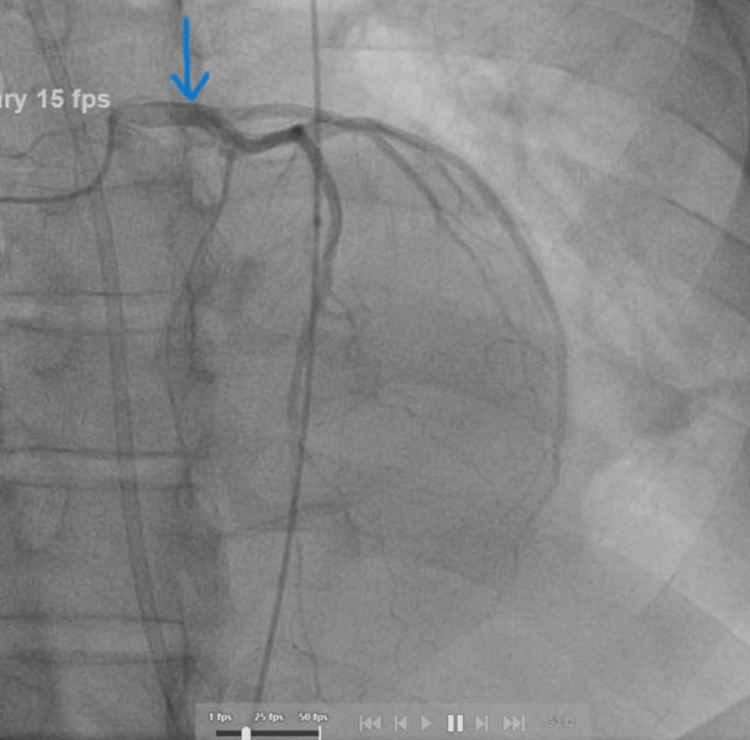
Coronary angiography showing flow in LAD artery with no obstruction/stenosis. LAD, left anterior descending

On echocardiography (done on admission day 2), the LV was dilated with normal wall thickness and severely depressed systolic function. As a result of global hypokinesis, LV relaxation was decreased during early diastole, resulting in an estimated left ventricular ejection fraction (LVEF) of 5%-10%. A chest X-ray revealed no evidence of pulmonary consolidation or effusion. In the following three days, coronary angiography was done showing no significant obstructive disease (Figures 2-3).

**Figure 4:**
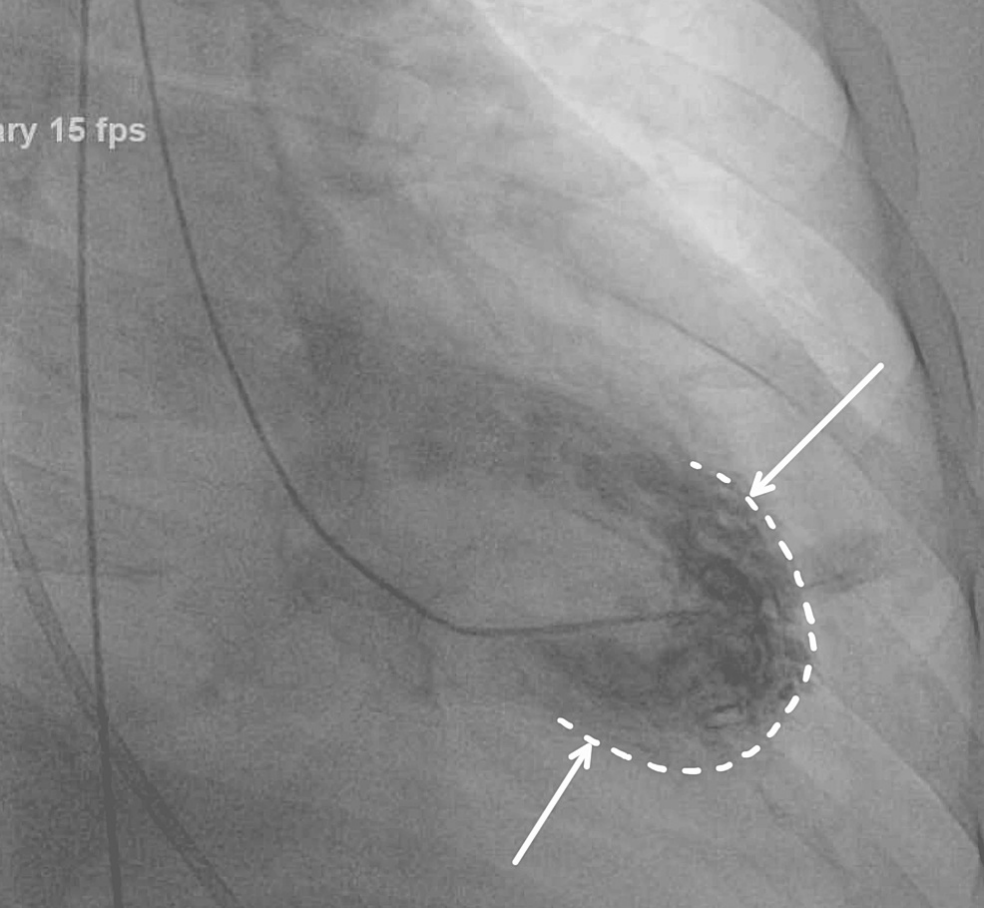
The left ventriculography showed mild dyskinesia at the apex with apical ballooning and a significant increase in contraction at the base depicting TCM (shown with white dotted line and arrows). TCM, Takotsubo cardiomyopathy

There was low right heart pressure, however, left ventricular end diastolic pressure (LVEDP) was elevated to 23 mmHg (normal range 4-12 mmHg). The left ventriculography showed mild dyskinesia at the apex and a significant increase in contraction at the base depicting TCM (Figure 4).

**Figure 5:**
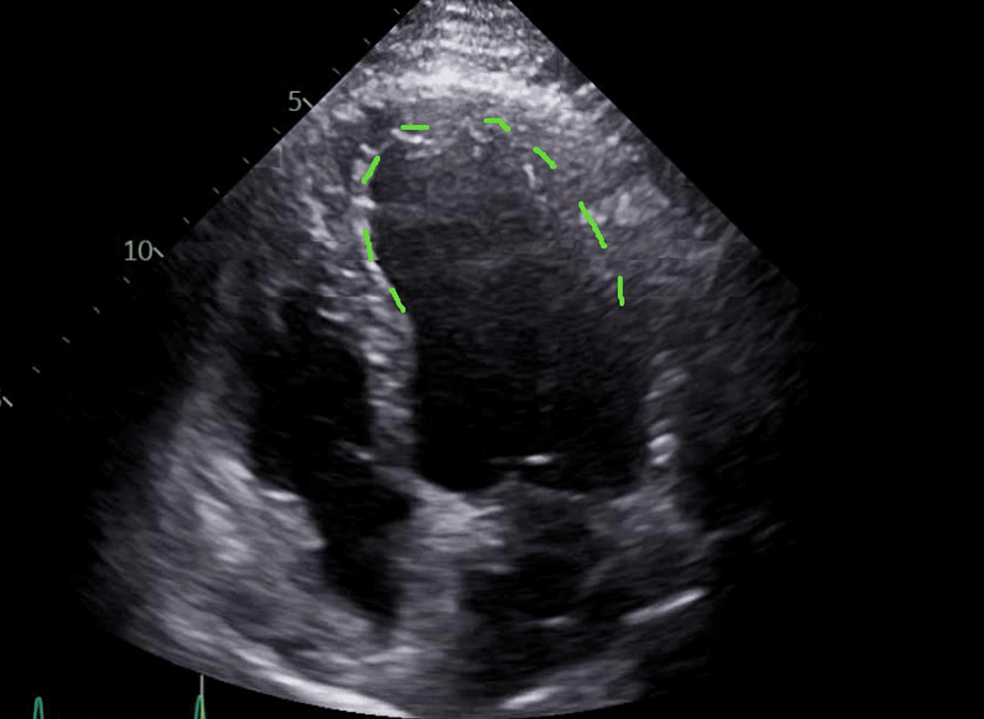
Echocardiographic findings suggestive of TCM with characteristic apical ballooning (shown with green dotted line). TCM, Takotsubo cardiomyopathy

A repeat Echo done three days later showed overall LV contractility improvement and an increased LVEF of about 30% with the apical ballooning characteristic of TCM (Figure 5).

**Figure 6:**
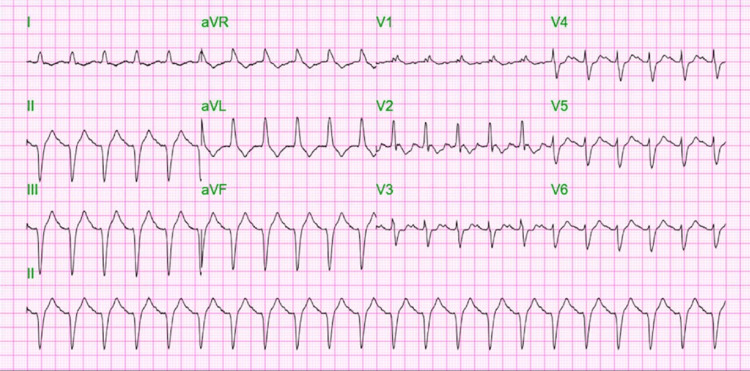
A repeat ECG demonstrated wide QRS tachycardia of the RBBB and LAFB that were not present on admission. ECG, electrocardiogram; RBBB, right bundle branch block; LAFB, left anterior fascicular block

A repeat ECG demonstrated wide QRS tachycardia of the right bundle branch block (RBBB) and left anterior fascicular block (LAFB) that were not present on admission and a complication of the resuscitation process (Figure 6).

Unfortunately, due to prolonged downtime, the patient remained in an irreversible comatose state following the CA and succumbed to cardiopulmonary failure and death due to cocaine overdose. 

## Discussion

Takotsubo cardiomyopathy, also known as “stress cardiomyopathy” or "broken heart syndrome," is characterized by reversible apical ballooning in the LV without demonstrable coronary artery obstruction [14-15]. “Tako-tsubo” in Japanese means “fishing pot for trapping octopus,” which has a round bottom and narrow neck similar to the appearance of the heart in TCM [14]. Lately, TCM nomenclature is changed to Takotsubo Syndrome (TTS) as it represents a wide array of emotional or physical triggers leading to LV dysfunction, therefore, best described as ‘syndrome’ [7]. There is a lack of worldwide consensus on formulating criteria for diagnosing TTS. Ghadri et al. developed the same for the identification and risk stratification of TTS [16]. It included (a) transient LV dysfunction (hypokinesia, dyskinesia, or akinesia) presenting as apical ballooning; (b) emotional or physical trigger with few societies excluding neurological as well as pheochromocytoma trigger; (c) new EKG abnormalities; (d) the moderate elevation of cardiac biomarkers; (e) significant CAD is not a contraindication; (f) primarily affecting postmenopausal females; and (g) no evidence of infectious myocarditis [16]. The modified Mayo Clinic diagnostic criteria include transient LV wall motion defect, no evidence of obstructive CAD, new EKG findings or elevated biomarkers, and no myocarditis [17].

About 90% of TCM cases are found in women, specifically postmenopausal women, with a mean age of 67-70 years and the risk is about 10 times higher in women than men [18-19]. Several studies have shown that TCM is associated with a history of depression, anxiety, and type-D personality because of an increased norepinephrine response to emotional stress and the presence of identifiable stressful triggers in two-thirds of cases [20]. Despite women having a greater predominance of developing TCM, men represent about 10% of cases and have worse outcomes with the data showing a comparison of in-hospital death from TCM from women being 3.8% and men being 7.3% [17]. The true incidence of TCM is currently unknown, but it is suspected that there are about 50,000-100,000 cases in the US per year with similar statistics for cases in Europe [21]. About 1%-3% of all acute coronary syndromes and 5%-6% of ST-segment elevation myocardial infarction (STEMI) in women have been eventually diagnosed as TCM [22]. Cocaine use is a rare cause of TCM with only a handful of cases documented [8-12]. Despite its rarity, cocaine-induced TCM is a significant concern due to its potential for serious morbidity and mortality.

Several pathophysiological mechanisms for the development of TTS have been proposed. The main mechanisms are autonomic nervous system dysfunction with sympathetic nervous system hyperactivation, myocardial ischemia, LV outlet obstruction, and blood-borne catecholamine surge [23-26]. The catecholamine hypothesis is possibly the most widely accepted pathophysiologic mechanism in TTS. Supraphysiological levels of catecholamine and neuropeptides (norepinephrine, epinephrine, and dopamine) stimulate beta-2 receptor coupling from Gs protein pathway to Gi protein pathway, leading to negative inotropy and resultant left ventricular contractile dysfunction [26-27]. The apex of the heart has the largest concentration of beta-adrenergic receptors, making it particularly susceptible to this surge resulting in typical findings of ballooning of the ventricle [28]. In the 1800s, cocaine was used as an anesthetic agent but is now classified as a Schedule II substance with a high abuse potential responsible for the most common cause of drug-related death [2]. Cocaine stimulates the sympathetic nervous system by directly inhibiting the reuptake of norepinephrine, dopamine, and serotonin with an increase in the sensitivity of adrenergic nerve endings to norepinephrine as a consequence of this stimulation of the central sympathetic outflow [3]. Cocaine toxicity can cause myocardial infarctions, myocardial ischemia, coronary vasospasms, arrhythmias, myocarditis, and cardiomyopathy [29]. Cocaine is ultimately thought to mimic sudden stress and induce a similar sequence of events resulting in microvascular spasm, myocardial stunning, or direct myocardial injury consistent with the diagnosis of TCM [30].

Our patient was brought to the hospital after CA likely cocaine-induced emphasizing that TTS might be a consequence of the stress associated with CA and/or CPR [31]. As per review done by Gili et al. the out-of-hospital CA was not associated with higher mortality than in-hospital CA [31]. It is difficult to assess and differentiate whether TTS is the cause or the consequence of CA, although the former explanation is more likely [32]. CA leads to LV dysfunction with global hypokinesia being the more frequent alteration leading to TTS-like patterns similar to our case presentation [33-34]. In most cases, TTS onset is associated with massive increases in circulating catecholamine levels causing myocardial stunning and edema which may lead to life-threatening arrhythmias [35]. The average in-hospital mortality rate for a patient with TCM who suffers a CA is 2.5% [36]. The correlation between cocaine use and the development of TCM involves several factors such as the time of presentation in the hospital, illegal use, short half-life of cocaine, the disparity in health care, and other cardiac comorbidities [27]. Further studies should be done to explore the statistics of mortality in CA at initial presentation with TTS as etiology, to emphasize efforts in prompt diagnosis and timely intervention.

Historically, TCM is considered a benign and reversible condition. It takes about two to three weeks for most TCM patients to recover, and their long-term prognosis is generally good [37]. Heart failure and pulmonary edema are the most common clinical complications [15]. CA is relatively common in TTS, typically occurs at the initial presentation, and is associated with worse outcomes [31]. Acute effects of cocaine on the cardiovascular system are well known with arrhythmias, myocardial infarction, coronary spasm, and myocarditis to name a few [2]. However, cocaine-induced TCM with CA as an intermediary condition is a rare presentation, especially in the male population [31]. The purpose of our case report is to raise awareness among physicians about this rare association so that appropriate measures/interventions done on time can reduce mortality in TCM.

## Conclusions

Takotsubo cardiomyopathy is a rare cardiovascular condition that has been implicated in the etiopathogenesis of sudden cardiac death and CA. Cocaine is a notorious drug for causing several cardiovascular side effects including coronary artery spasms, myocarditis, arrhythmias, and congestive heart failure. Association of cocaine use with subsequent development of TCM/TTS is a rare presentation. As suggested by our case report, TCM and catecholamine surge have a well-established relationship with cocaine mimicking stress and leading to adverse cardiac events like CA. There are no standardized recommendations for pharmacological treatment and prevention of episodes of TCM and further studies/trials need to be done to answer questions regarding the clinical diagnostic criteria, etiology, pathophysiology, and management of this syndrome.
